# Bee venom effects on ubiquitin proteasome system in hSOD1^G85R^-expressing NSC34 motor neuron cells

**DOI:** 10.1186/1472-6882-13-179

**Published:** 2013-07-18

**Authors:** Seon Hwy Kim, So Young Jung, Kang-Woo Lee, Sun Hwa Lee, MuDan Cai, Sun-Mi Choi, Eun Jin Yang

**Affiliations:** 1 Department of Acupuncture & Moxibustion, Korea Institute of Oriental Medicine, 483 Expo-ro, Daejeon, Yuseong-gu 305-811, Republic of Korea; 2 Department of Medical Research, Korea Institute of Oriental Medicine, 483 Expo-ro, Daejeon, Yuseong-gu 305-811, Republic of Korea

**Keywords:** hSOD1^G85R^, Ubiquitin proteasome system (UPS), Bee venom (BV), Amyotrophic lateral sclerosis (ALS), NSC34 motor neuronal cells

## Abstract

**Background:**

Amyotrophic lateral sclerosis (ALS) is a neurodegenerative disease that results from a progressive loss of motor neurons. Familial ALS (fALS) is caused by missense mutations in Cu, Zn-superoxide dismutase 1 (SOD1) that frequently result in the accumulation of mutant protein aggregates that are associated with impairments in the ubiquitin-proteasome system (UPS). UPS impairment has been implicated in many neurological disorders. Bee venom (BV) extracted from honey bees has been used as a traditional medicine for treating inflammatory diseases and has been shown to attenuate the neuroinflammatory events that occur in a symptomatic ALS animal model.

**Methods:**

NSC34 cells were transiently transfected with a WT or G85R hSOD1-GFP construct for 24 hrs and then stimulated with 2.5 μg/ml BV for 24 hrs. To determine whether a SOD1 mutation affects UPS function in NSC34 cells, we examined proteasome activity and performed western blotting and immunofluorescence using specific antibodies, such as anti-misfolded SOD1, anti-ubiquitin, anti-GRP78, anti-LC3, and anti-ISG15 antibodies.

**Results:**

We found that GFP-hSOD1^G85R^ overexpression induced SOD1 inclusions and reduced proteasome activity compared with the overexpression of GFP alone in NSC34 motor neuronal cells. In addition, we also observed that BV treatment restored proteasome activity and reduced the accumulation of ubiquitinated and misfolded SOD1 in GFP-hSOD1^G85R^-overexpressing NSC34 motor neuronal cells. However, BV treatment did not activate the autophagic pathway in these cells.

**Conclusion:**

Our findings suggest that BV may rescue the impairment of the UPS in ALS models.

## Background

Amyotrophic lateral sclerosis (ALS) is the most common adult-onset motor neurodegenerative disease. ALS is characterized by a progressive loss of motor neurons in the spinal cord, brain stem and motor cortex that leads to the rapid and progressive atrophy of skeletal muscles. Although the majority of ALS cases are sporadic (sALS), 5–10% are inherited autosomal-dominant cases (fALS) that result from the generation of misfolded or abnormal proteins [1]. Among fALS cases, approximately 15–20% involve missense mutations in the gene encoding copper/zinc superoxide dismutase (SOD1). Although many studies have demonstrated that oxidative stress, mitochondrial degeneration, cytoskeletal alterations, and growth factor abnormalities occur in ALS models, the mechanism of pathogenicity in ALS is completely unknown. One hypothesis suggests that mutated and misfolded SOD1 is a critical factor, and these proteins are believed to play a major role in motor neuron dysfunction and death in ALS [2].

Abnormal and misfolded proteins are degraded by the ubiquitin-proteasome system (UPS) and autophagy-lysosome pathway (ALP). Impairments in these systems may lead to the accumulation and aggregation of misfolded proteins, resulting in cellular toxicity and death. Wild-type SOD1 and the A4V, G85R and G93A mutant SOD1 proteins are degraded by both the UPS and autophagy degradation pathways [3]. The A4V, G85R and G93A mutant SOD1 proteins are more easily misfolded and more unstable than wild-type SOD1 [3-5]. For example, inhibiting proteasomes in culture using epoxomicin induced the accumulation of mutant hSOD1^G93A^[3]. Furthermore, misfolded or ubiquitinated SOD1 is found in the motor neurons of a transgenic mouse model of ALS [6]. The autophagic degradation of wild-type and mutant SOD1, however, was blocked in cultures treated with 3-MA, an inhibitor of macroautophagy, and ammonium chloride, an inhibitor of lysosomal proteolysis, suggesting that autophagy contributes to the clearance of wild-type and mutant SOD1 [3,7]. *In vivo* studies have demonstrated that macroautophagy plays an important role in preventing neurodegeneration in mice [8,9]. In addition to the UPS, mutant SOD1 is also cleared by autophagy. This finding supports the hypothesis that the impaired autophagic degradation of mutant SOD1 is an important mechanism leading to neurodegeneration in ALS and that the misfolding of mutant SOD1 is a critical feature of ALS pathology. Therefore, reducing the accumulation of misfolded or aggregated SOD1 may be of therapeutic value for treating ALS.

Bee venom (BV) extracted from honey bees has been used in traditional oriental medicine. BV contains a variety of peptides, including melittin, apamin and adolapin, as well as enzymes and biogenic active amines. Recent studies have reported that BV has anti-nociceptive [10,11] and anti-inflammatory [12,13] effects. It has been used to treat disease models for rheumatoid arthritis [11] and cancer [14] as well as neurodegenerative disease models, such as Alzheimer’s disease (AD) [15], Parkinson’s disease (PD) [16], and ALS [17].

In this study, we investigated the effects of BV on proteasome activity in cells expressing the mutant hSOD1^G85R^ gene and demonstrated that BV restored proteasome activity, resulting in a reduction in ubiquitinated mutant hSOD1^G85R^ in NSC34 motor neurons. Furthermore, we showed that BV treatment significantly reduced the amount of misfolded SOD1 in hSOD1^G85R^-expressing NSC34 cells. The findings of this study suggest that BV treatment may be able to eliminate the cell toxicity induced by ubiquitinated or misfolded mutant hSOD1^G85R^ in motor neurons and restore proteasome activity in ALS.

## Methods

### Plasmid constructs

The pcDNA3-GFP expression plasmid containing GFP-tagged wild-type or G85R-mutant SOD1 were kindly donated by Yoshiaki Furukawa (RIKEN Brain Science Institute).

### Cell culture and transfections

The motor neuron cell line NSC34 was purchased from Cellutions Biosystems Inc. (Toronto, ON, Canada) and maintained in Dulbecco’s modified Eagle’s medium (DMEM) supplemented with 10% fetal bovine serum (Gibco, NY, USA), 100 U/ml penicillin (Gibco, NY, USA), and 100 μg/ml streptomycin (Gibco, NY, USA) at 37°C in 5% CO_2_, as recommended by Cellutions Biosystems Inc. Each vector was transiently transfected into NSC34 cells using Lipofectamine 2000 (Invitrogen, NY, USA). Approximately 80% of plated cells were transfected using our experimental procedures. For BV (Sigma, MO, USA) treatment, BV solubilized with normal saline treated with transfected cells by 2.5 μg/ml.

### Proteasome activity assay

Cells were lysed using RIPA lysis buffer (50 mM Tris–HCl pH 7.4, 1% NP-40, 0.1% SDS, and 150 mM NaCl) and the Complete Mini Protease Inhibitor Cocktail (Roche, Basel, Switzerland) and centrifuged at 14,000 rpm at 4°C for 20 min. The supernatant fraction was collected in a new tube, and the protein concentration was determined using a BCA protein kit (Interchim, Paris, France). The proteasomal activity was measured using the 20S Proteasome Activity Assay kit according to the manufacturer’s instructions (Chemicon Inc., CA, USA). The fluorescence of each sample was evaluated with a spectrofluorometer at excitation and emission wavelengths of 370 nm and 430 nm, respectively. The level of proteasomal activity was determined from the increase in the fluorescence of the reaction products.

### SOD1 aggregation assay

For the SOD1 aggregation assay, 5 × 10^5^ NSC34 cells were plated on a cover slip and transfected using Lipofectamine 2000 (Invitrogen, NY, USA) for 48 hrs. The cells were fixed with 4% paraformaldehyde for 10 min at room temperature and then washed two times with phosphate-buffered saline. The cover slip was fixed on a glass slide using Fluoromount containing DAPI. Transfected cells were observed using an Olympus fluorescent microscope (Olympus, Tokyo, Japan) under identical exposure settings.

For quantification of aggregated cells, 10 microscopic visual fields were randomly selected and aggregated cells including scattered filamentous or condensed forms among GFP-positive cells were counted. The % aggregation was the percentage of aggregated cells among the GFP-positive cells which transfected cells.

### Immunoblotting and immunoprecipitation

Cell lysates were extracted using ice-cold lysis buffer containing 50 mM Tris HCl pH 7.4, 1% NP-40, 0.1% SDS, 150 mM NaCl, and the Complete Mini Protease Inhibitor Cocktail (Roche, Basel, Switzerland). The protein concentration was determined using a BCA Protein Assay Kit (Pierce, IL, USA). Total cell lysates were immunoprecipitated with an anti-GFP antibody overnight. After incubating with protein A/G plus agarose beads, the immunocomplex-beads were washed with RIPA buffer. Boiled cell lysates (20 μg total protein per lane) or immunocomplexes were subjected to SDS-polyacrylamide gel electrophoresis (SDS-PAGE) and transferred to nitrocellulose membranes (Whattman, Kan, USA). The membranes were blocked in blocking solution (5% skim milk) and incubated with primary antibodies against the following proteins: *α*-tubulin (1:5000, Abcam, MA, USA), GFP, GRP78 (1:1000, Santa Cruz Biotec, CA, USA), ISG15 (1:500, Santa Cruz Biotec, CA, USA), LC3 (1:1000, sigma, MO, USA), and ubiquitin (1:2,000, DAKO, Denmark). The membranes were incubated with a horseradish peroxidase (HRP)-conjugated secondary antibody for 2 hrs at room temperature. Specific protein bands were detected with the SuperSignal West Femto Chemiluminescent Substrate (Pierce, IL, USA) or enhanced chemiluminescence reagents (Amersham Pharmacia, NJ, USA). *α*-tubulin was used as an internal control for protein loading. Protein bands were detected and analyzed using a FusionSL4-imaging system. Quantification of the immunoblotted bands was performed using the Bioprofil (Bio-1D version 15.01, Viber Lourmart).

### SOD1 polymerization

To analyze SOD1 polymerization, 15 mg of fresh cells was homogenized in 200 μl homogenizing buffer (50 mM HEPES, 1 mM EDTA, 100 mM idoacetamide), incubated at 37°C for 1 hrs and then centrifuged at 20,000 g for 5 min. The pellets were washed three times with a washing buffer (50 mM HEPES, 1 mM EDTA, 100 mM idoacetamide and 0.1% Nonidet P-40, pH 7.2) and centrifuged at 20,000 g for 10 min. The final pellet (the insoluble fraction) was suspended in 200 μl of final solution A (50 mM HEPES, 1 mM EDTA, 100 mM idoacetamide, and 2.5% sodium dodecyl sulfate (SDS), pH 7.2). The insoluble fraction of each sample was either treated with β-mercaptoethanol or left untreated before boiling and then run through a 15% SDS polyacrylamide gel for western blot analysis using an antibody against misfolded hSOD1 (Medimabs, Quebec, Canada).

### Statistical analysis

For the comparisons between experimental groups, the significant differences were analyzed by one-way ANOVA using the PRISM program (GraphPad). The data are expressed as the means ± S.E.M; p < 0.05 was considered statistically significant.

## Results

### BV treatment restored UPS function in hSOD1^G85R^-expressing NSC34 motor neuron cells

**Figure 1:**
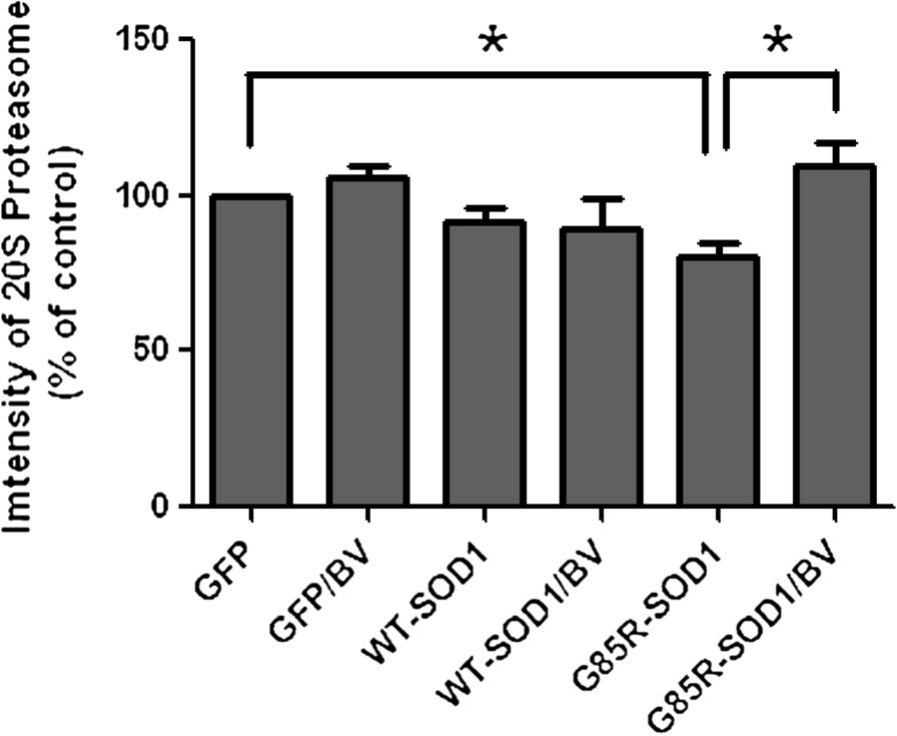
**BV treatment restores proteasome activity.** NSC34 cells (2 × 10^6^) were transfected with GFP, GFP-hSOD1 or GFP-hSOD1^G85R^ constructs for 48 hrs and then treated with 2.5 μg/ml BV or equal volume of saline for 24 hrs prior to being lysed. The proteasome activity of the lysates was assayed using the 20S Proteasome Activity Assay kit. Proteasome activity was significantly decreased in NSC34 cells that transiently overexpressed the mutant G85R SOD1 compared with the control. BV treatment increased proteasome activity. The values shown are the means ± SEM of data obtained from three independent experiments. * p < 0.05. BV; bee venom.

A previous study showed that wild-type and mutant hSOD1 were degraded by macroautophagy and the proteasome [3]. To investigate whether the overexpression of hSOD1^G85R^ in NSC34 motor neuron cells affects proteasome activity, 20S proteasome activity was examined. Consistent with previous studies, hSOD1^G85R^ overexpression in NSC34 cells suppressed proteasome activity compared with GFP-transfected NSC34 cells (Figure 1). However, treatment with 2.5 μg/ml BV for 24 hrs significantly restored proteasome activity in hSOD1^G85R^-overexpressing NSC34 motor neuron cells (Figure 1). These data suggest that BV treatment improved the function of the UPS in this mutant ALS cell model.

### BV treatment reduced the formation of ubiquitinated and misfolded hSOD1^G85R^ in NSC34 motor neuron cells

**Figure 2:**
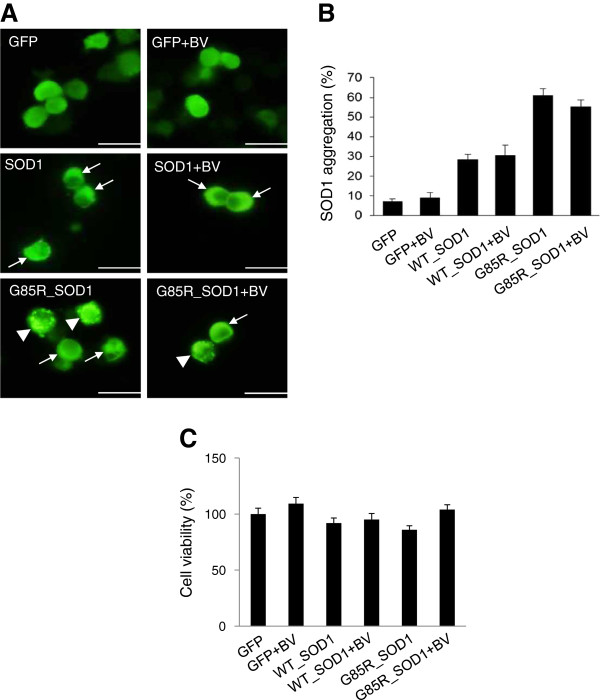
**BV treatment does not significantly reduce the aggregation of SOD1 protein. (A)** Cells were transiently transfected with a WT or G85R hSOD1-GFP construct for 24 hrs and then treated with 2.5 μg/ml BV or equal volume of saline for 24 hrs. At 48 hrs post-transfection, the cells were fixed and immunofluorescent staining was performed with an anti-GFP primary antibody. GFP-SOD1 aggregation was observed under a fluorescence microscope with fluoromount containing DAPI. Arrowheads indicate scattered filamentous aggregates, and arrows indicate condensed aggregates. **(B)** Quantification of A. The values shown are the means ± SEM of data obtained from three independent experiments. (bars, 25 μm.) * p < 0.05, ** p < 0.01. **(C)** Cells transfected with a WT or G85R hSOD1-GFP for 24 hrs treated with 2.5 μg/ml BV or equal volume of saline for 24 hrs. Cell viability was measured with MTT assay.

The overexpression of both wild-type and ALS-causing, mutant hSOD1 led to the production of aggregates [18,19]. To determine whether BV treatment affects the aggregate formation induced by mutant hSOD1^G85R^ overexpression, we counted the number of aggregates formed in the presence or absence of BV treatment for 48 hrs after transfection with either GFP-tagged wild-type or G85R mutant hSOD1 in NSC34 motor neuron cells. We found that BV treatment reduced the number of aggregates formed as a result of hSOD1^G85R^ overexpression but that this reduction was not statistically significant (Figure 2). Next, we investigate whether BV treatment affect cell death by hSOD1^G85R^ overexpressed-aggregates in NSC34 cells. As shown in Figure 2C, we did not observe significant cell death in wild-type and G85R over expressed neuronal cells.

**Figure 3:**
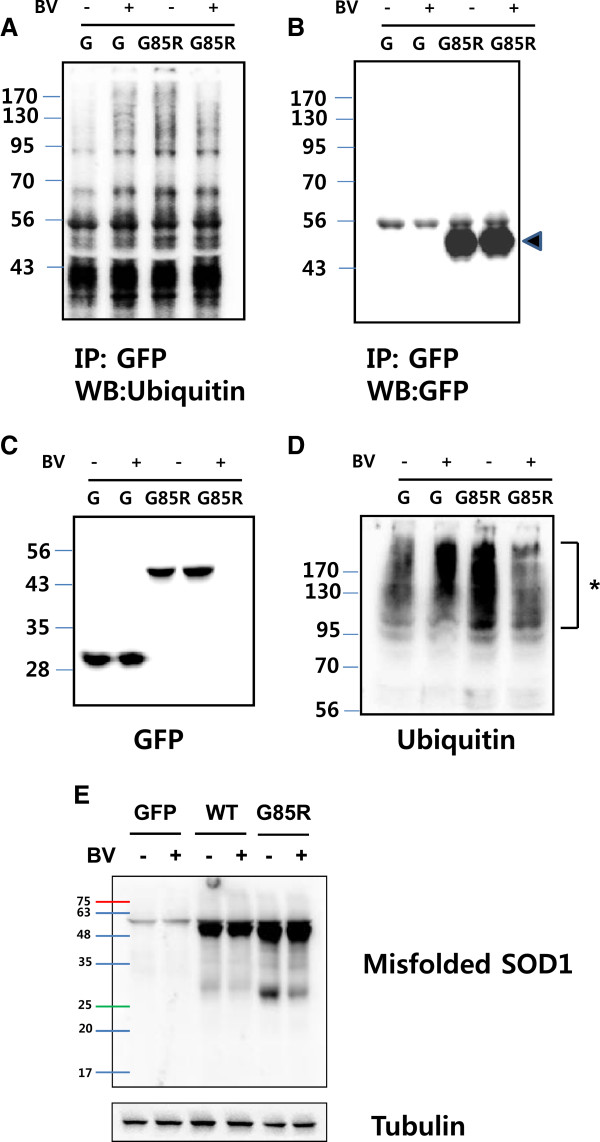
**BV treatment reduces the accumulation of ubiquitinated and misfolded SOD1 proteins.** After the transient transfection of NSC34 motor neuron cells with GFP or GFP-tagged hSOD1^G85R^ for 24 hrs, cells were treated with 2.5 μg/ml BV or equal volume of saline for 24 hrs or left untreated. The cells were lysed using RIPA buffer, immunoprecipitated with anti-GFP and then western blot analysis was performed using primary anti-ubiquitin **(A)** and anti-GFP **(B)** antibodies. The arrowhead indicates GFP-tagged mutant hSOD1^G85R^. Total cell lysates were analyzed by western blotting with anti-GFP (**C**) and anti-ubiquitin **(D)** primary antibodies. Asterisks indicate ubiquitinated proteins. To detect misfolded SOD1, NSC34 cells transfected with wild-type or hSOD1^G85R^ constructs were lysed using the SOD1 polymerization method described previously, and an immunoblot analysis was performed with an anti-misfolded SOD1 antibody **(E)**. The membranes were reprobed with an anti-tubulin antibody as a loading control. G: GFP-transfected cells.

To confirm the effect of BV on aggregates resulting from hSOD1^G85R^ overexpression, we investigated the formation of ubiquitinated or misfolded hSOD1^G85R^ in NSC34 cells in the presence or absence of BV treatment. As shown in Figure 3A and D, the formation of ubiquitinated hSOD1^G85R^ and total ubiquitinated protein levels were significantly reduced in BV treated hSOD1^G85R^-overexpressing NSC34 motor neuron cells. Furthermore, we also found that misfolded SOD1 levels were reduced in these cells. These findings strongly suggest that mutant SOD1 aggregate formation is correlated with a reduction in proteasome activity and that BV treatment prevents the misfolding of hSOD1^G85R^ proteins and ameliorates the impairments in UPS activity in mutant hSOD1-expressing motor neuron cells.

### BV does not activate the autophagic signaling pathway

**Figure 4:**
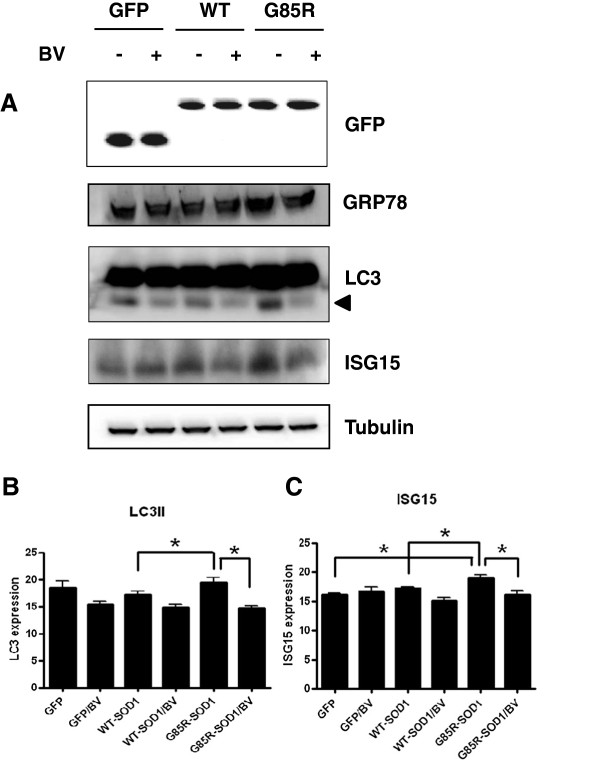
**BV treatment does not activate the autophagic signal pathway.** NSC34 cells were transiently transfected with the GFP control vector or GFP-tagged wild-type hSOD1 or mutant hSOD1^G85R^. Approximately 24 hrs after transfection, the cells were treated with 2.5 μg/ml BV or equal volume of saline for 24 hrs or left untreated. The cells were lysed using RIPA buffer, and western blot analysis was performed with anti-GRP78, anti-LC3, and anti-ISG15 antibodies to examine the expression of autophagic-degradation proteins **(A)**. The arrowhead indicates LC3II protein levels. The membranes were reprobed with an anti-α-tubulin antibody as a loading control. Quantification of LC3II **(B)** and ISG15 **(C)** are shown. The values shown are the means ± SEM of data obtained from three independent experiments. * p < 0.05, ** p < 0.01.

Next, we investigated whether BV enhances the autophagy signal pathway to eliminate the aggregates and misfolded hSOD1^G85R^ proteins in NSC34 cells. As shown in Figure 4, hSOD1^G85R^ overexpression induced the expression of the microtubule-associated protein 1 light chain 3 II (LC3II) and ISG15 proteins compared with the overexpression of GFP or wild-type hSOD1 in NSC34 cells. However, BV treatment significantly reduced the expression of autophagosome-related proteins, including LC3II and ISG15 relative to untreated hSOD1^G85R^ overexpression in NSC34 motor neuron cells. This result indicates that BV treatment improves proteasome activity but not autophagosome activation in mutant-hSOD1-overexpressing motor neuron cells.

## Discussion

ALS is often considered a proteinopathy because the generation of misfolded or abnormal proteins with the propensity to aggregate and accumulate in damaged neurons is a common feature of both sporadic ALS (sALS) and familial ALS (fALS) [6,20]. This study reveals that BV treatment restores the proteasome activity inhibited by hSOD1^G85R^ expression and reduces the levels of misfolded and ubiquitinated mutant SOD1 and aggregates in NSC34 motor neuron cells. BV treatment, however, did not affect the autophagy endolysosomal pathway in hSOD1^G85R^-expressing motor neuron cells.

BV has been used in oriental medicine to reduce inflammation in rheumatoid arthritis and for the relief of pain [21]. BV is composed of a variety of bio-active enzymes, different peptides and proteins, including melittin, apamin, adolapin, mast cell degranulating peptide and phospholipase A2 [22]. Melittin is a major component of BV, and Yang et al. demonstrated that the administration of melittin in an ALS animal model improved proteasome activity and attenuated neuroinflammation in the spinal cord and brainstem [23]. A previous study reported that BV inhibits growth and exhibits cytotoxic effects in hepatocellular carcinoma cells [24], and another study suggests that BV induces pain and hyperalgesia [25]. Another study, however, reported that BV reduces inflammatory reactions and enhances antinoceptive effects [26]. Furthermore, BV has a protective role against 1-methyl-4-phenyl-1,2,3,6-tetrahydropyridine (MPTP)-induced apoptosis in neuronal cells [27] and diminishes neuroinflammation in an MPTP-induced Parkinson animal model and an hSOD1^G93A^-expressing ALS animal model [17,28].

Abnormally modified proteins and proteinaceous inclusions can be found in most fALS and sALS cases. Moreover, these modified proteins are often components of the inclusions in the motor neurons of not only patients but also animal and cell models of the disease [29-31]. UPS, which is the main intracellular proteolytic system, and the autophagy-lysosome pathway are the predominant mechanisms used to eliminate abnormal proteins and to degrade cytoplasmic proteins; this degradation is mediated by macroautophagy [32]. In ALS, mutant SOD1 is degraded by proteasomes [33,34], and the partial inhibition of proteasome activity induces the formation of large SOD1-containing aggregates [35,36]. When the proteasomes are inhibited, the aggregates that significantly increase in size and abundance become ubiquitinated, and this is accompanied by the formation of high-molecular-weight proteins that represent the dimeric/oligomeric forms of misfolded mutant SOD1 [37].

Changes in proteasome activity and the accumulation of ubiquitinated protein aggregates have been demonstrated in ALS patients and animal models [5,38,39]. In this study, we found that the expression of mutant hSOD1^G85R^ reduced proteasome activity, which is consistent with the findings of previous studies (Figure 1). However, we did not observe that cell death by overexpressing hSOD1^G85R^ in NSC34 motor neuron cells (Figure 2). Interestingly, BV treatment enhanced proteasome activity in GFP-hSOD1^G85R^-expressing motor neuron cells compared with cells transfected with GFP alone (Figure 1). Furthermore, we found that BV treatment reduced the amount of ubiquitinated mutant SOD1 and misfolded SOD1 in hSOD1^G85R^-expressing NSC34 motor neuron cells (Figures 2 and 3).

However, BV consists of many enzymes, peptides and proteins and melittin is a major component of BV. In previous study, we found that melittin treatment improved proteasome acitivity in hSOD1^G93A^ transgenic mice [23]. Therefore, we suggest that melittin from BV may be a bioactive component in the improvement of proteasome activity in GFP-hSOD1^G85R^-expressing motor neuron cells.

The ubiquitination and sumoylation of proteins are mechanisms by which proteins are selectively targeted for degradation, and this specificity is believed to be necessary for the maintenance of cellular homeostasis [40]. Protein sumoylation and ubiquitination are complex processes. In many cases, including those in which a single lysine is targeted, the two modifications do more than simply antagonize or compete with each other [41]. The ubiquitination and sumoylation pathways often compete with each other because the same lysine residue is frequently used in both of their substrates. For example, sumoylation of the pathogenic huntingtin fragment prevents ubiquitination at the same lysine residue, which abrogates proteasome-mediated degradation [42]. In this study, we did not detect a significant reduction in the number of SOD1-induced aggregates in BV-treated hSOD1^G85R^-expressing NSC34 motor neuron cells (Figure 2), even though the BV treatment decreased the formation of ubiquitinated SOD1 (Figure 3). These results suggest that the mutant SOD1 aggregates may contain sumoylated SOD1. Furthermore, Fei E et al. have shown that hSOD1^G85R^ sumoylation increases and may enhance the stability of SOD1 [43]. It has been reported that the sumoylation of the lysine residue of some proteins blocks ubiquitination at the same site, which inhibits protein degradation by the proteasome and alters protein function [42,44].

Misfolded SOD1 has a toxic gain of function including generation of reactive oxygen and nitrogen species [45], and induction of mitochondrial dysfunction [46]. Therefore, the research on consequent molecular pathway mediated by misfolding SOD1 would be valuable for finding the effective treatment for ALS patients.

Growing evidence supports a role for the autophagy endolysosomal pathway in the pathogenesis of ALS. Moreover, the accumulation of autophagosomes was observed in the spinal cords of sporadic ALS patients [47], indicating the presence of autophagic dysfunction in ALS. Autophagic dysfunction results from defects in the initiation (formation of autophagosomes) and/or maturation stages of the autophagic process or an imbalance between these stages. These conditions result in the aberrant accumulation of misfolded and/or aggregated proteins within the cells. In this study, BV treatment did not activate the autophagic lysosomal pathway in hSOD1^G85R^-expressing NSC34 motor neuron cells (Figure 4). This finding suggests that BV treatment reduces the aggregation of proteins specifically through ubiquitination and not activation of the autophagy pathway.

It has been well established that cases of ALS associated with mutations in SOD1 are caused by the toxic properties associated with the propensity of mutant SOD1 to misfold [48,49]. Ubiquitinated, insoluble aggregates are the pathological hallmark of several neurodegenerative diseases, including ALS, PD and AD. The propensity for aggregate formation associated with mutant SOD1 proteins, and the resulting toxic features, may be related to the phenotypic expression of the disease [2].

Currently, it is believed that a complex network incorporating multiple toxicity pathways, rather than a single independent mechanism, is involved in the pathogenesis of ALS. Pathogenic mechanisms that disturb protein homeostasis are of particular interest because the accumulation of insoluble protein aggregates is the cardinal pathological feature of ALS and other neurodegenerative diseases [50]. Although it remains to be determined whether such protein aggregates have a toxic or protective role in the pathogenesis of ALS, it is possible that the formation of aggregates results from an imbalance between the generation and degradation of misfolded proteins within neuronal cells. Therefore, the solubility of hSOD1^G85R^ aggregates should be investigated to determine whether insoluble proteins are associated with cell death in motor neuron cells.

## Conclusions

In this study, we have demonstrated the effects of BV on the UPS impairments caused by hSOD1^G85R^ overexpression in motor neuron cells. The reduction in proteasome activity may increase the number of mutant SOD1-positive aggregates and induce SOD1 misfolding in mutant hSOD1-expressing motor neuron cells.

BV treatment reduced the amount of ubiquitinated and misfolded SOD1 in an *in vitro* ALS model. Although little is known about the mechanisms by which BV affects proteasome regulation, the findings of this study suggest that BV should be considered as a candidate therapeutic agent for regulating ALS pathological events.

## Competing interests

The authors declare that they have no competing interests.

## Authors’ contributions

EJY designed the experiments and analyzed the data as well as edited the manuscript. SHK executed biochemical study and drafted the manuscript. SYJ and KWL participated in SOD1 aggregation assay and performed statistical analyses. SHL and MDC carried out plasmid preparation and proteasome acitivity. SMC discussed with the manuscript. All authors have read and approved the final manuscript.

## Pre-publication history

The pre-publication history for this paper can be accessed here:

http://www.biomedcentral.com/1472-6882/13/179/prepub
